# Short communication: Birth Shock!

**DOI:** 10.1016/j.puhip.2023.100371

**Published:** 2023-02-25

**Authors:** Susan Hogan, Rebecca Ashley

**Affiliations:** a Arts & Health, College of Arts, Humanities & Education, United Kingdom; b Institute of Mental Health, University of Nottingham, United Kingdom; c The Stillbirth and Neonatal Death Charity, London, United Kingdom

**Keywords:** Arts and health, Maternity and arts, Women's health, Birth trauma and art, Art and wellbeing, Perinatal arts, Health humanities

## Abstract

*Birth Shock!* is an AHRC-funded (AH/K003364/1) engagement-focused project exploring and enhancing the impact and reach of The Birth Project (AH/V000926/1). This study is particularly concerned with the role the arts can play in the perinatal period, especially in supporting the wellbeing of new mothers. In particular, we wished to assess the role of a suite of films exploring this subject with trainee and health-professional audiences.

## 1 Objectives

Within *The Birth Project*, the principal aims of the research focused on using the arts to interrogate birth discourses, and to challenge embedded assumptions. *The Birth Project* focused on four central questions.

● What role might arts engagement have to play in antenatal and postnatal care?
● To what extent are hospital practices, that are [potentially] iatrogenic in nature, implicated in postnatal distress?
● To what extent is ‘mutual recovery’ possible through engagement with the arts, and if so, to establish what form this may take?
● What, in particular, does an arts-based approach offer in exploring birth experiences and the transition to motherhood?

These questions were examined in a suite of films. This current research is an AHRC-funded, engagement-focused project exploring and enhancing the impact and reach of The Birth Project (AH/V000926/1) by engaging with new non-academic and trainee professional audiences. This aim has been primarily conducted through online and in-person screening workshops of *The Birth Project* films (AH/K003364/1). An objective has included soliciting feedback on the effectiveness of these resources in facilitating enhanced understanding of these topics via a Likert questionnaire and free-text box. We have grouped the Likert responses under *The Birth Project* original questions to present an overview of the effectiveness of the resources in relation to these questions.

The films are available via links from *The Birth Project* website & directly from *YouTube* and were used in the films screening workshops.

❖ *Creative Practice as Mutual Recovery. Visual Methodologies.* 2013. Susan Hogan Executive Producer, Sheffield Vision. 20 mins.
❖ *Mothers Make Art.* 2015. Susan Hogan Executive Producer. Sheffield Vision. 41 mins.
❖ *Arts Elicitation with New Mothers.* 2015. Susan Hogan Executive Producer. Sheffield Vision. 24 mins.
❖ *Birth Professionals Make Art.* 2015. Susan Hogan Executive Producer. Sheffield Vision. 30 mins.
❖ *Mothers Make Contemporary Art.* 2017. Susan Hogan Executive Producer. Sheffield Vision. 30 mins.
❖ *Birth Shock. The Documentary.* 2018. Susan Hogan Executive Producer. Sheffield Vision. 30 mins.

Two further shorter films have been produced with classroom use in mind.

❖ *Towards a Better Birth.* 2020. Susan Hogan Executive Producer. Co-edited with Eve Wood, Sheffield Vision. 8 mins.
❖ *If I were a Better Mother.* 2020. Susan Hogan Executive Producer. Co-edited with Eve Wood, Sheffield Vision. 8 mins.
❖ *Mothers Make Art.* 2021. Susan Hogan Executive Producer. Sheffield Vision. 41 mins. Version with French Subtitles.

## 2 Methods

*Birth Shock!* involved freely promoting the suite of films to reach non-academic audiences via the internet and at events and engagement activities, including 28 film-screening workshops, a symposium, film festivals and film screenings impact events. In addition to the film-screenings aimed at the general public, a total of 496 participants took part in a structured *Birth Shock* film-screening workshop during the course of the project. We received a total of 208 responses to our online survey, which equates to a response rate of 42% of all workshop participants.

Film workshops varied between 30 min and 3 h depending on the requirements of the host organisations. All workshops followed a structured format in which participants were briefed on the Birth Project research, viewed one to two of the project films, were invited to answer an anonymous feedback survey, and then engaged in a facilitated group discussion. Local debriefing contacts and online resources were provided to all participants. The main films focused on during the screening workshops were *Mothers Make Contemporary Art, Arts Elicitation with New Mothers*, and *Birth Professionals Make Art* [2].

### 2.1 Brief summary of qualitative data

● **What role might arts engagement have to play in antenatal and postnatal care?**

**Figure 1:**
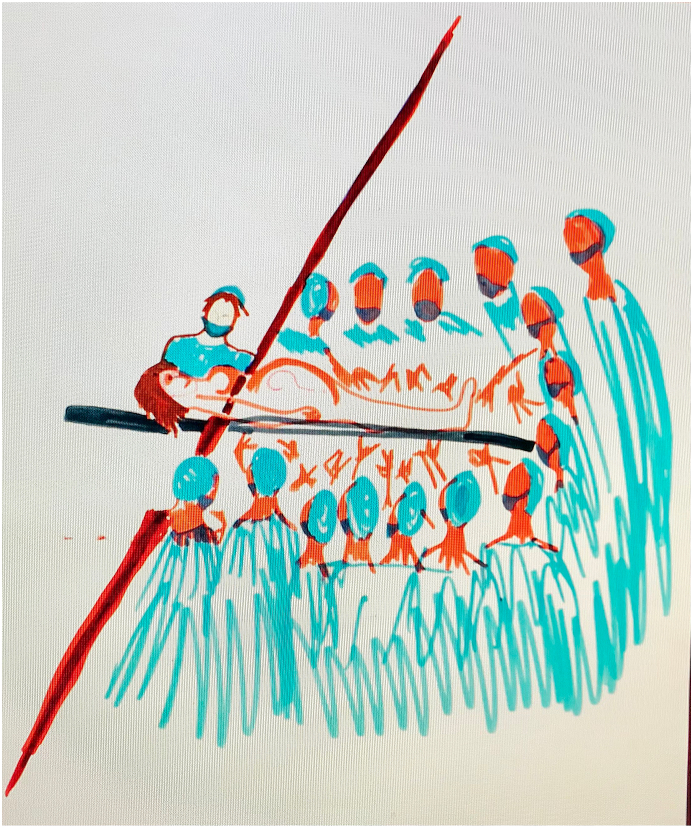
Impact image.

This question is explored in the films themselves, especially the longer films and the response that came out strongly, is that art making, as an act of volition, was invigorating and empowering, even to women feeling disempowered because of what had happened to them (see Fig. 1). Secondly, the use of art materials can *reveal* in unexpected ways, so art making can be an act of revelatory self-discovery for participants. Project publications explore these processes in some depth (Hogan 2015 [1]; Hogan 2019 [3]; Hogan 2020 [4]. The Likert survey data is not very focussed on the *process* of art making *per se*. However, a number of workshop participants did comment on how the films had made them more aware of the use of art as an enabler, which is a good general outcome.

● **To what extent are hospital practices, that are [potentially] iatrogenic in nature, implicated in post-natal distress?**

In reflecting on this question, we considered the responses workshop participants provided surrounding their feelings and assumptions about hospital practices and postnatal distress. In the survey feedback, participants overwhelmingly reported that they either somewhat agreed (44.6%), or strongly agreed (43.6%) that hospital practices were implicated in postnatal distress. Within the range of mean scores by professional group, midwives (4.4), peer support workers (4.3), student midwives (4.2), and medical students [4] were most likely to agree with this statement. Health visitors (3.4) felt less confident that hospital practices were implicated in postnatal distress.

Workshop participants, across a range of professional backgrounds, reflected on their increased awareness of the ways in which hospital-based practices and clinical procedures could be implicated in postnatal distress. Several comments by students pointed to a specific increased awareness of the longevity of postnatal distress. Participants reported that the films shed new light on the ways in which parents might be continuing to process their birth experiences long after their intervention as a professional had concluded.

● **To what extent is ‘mutual recovery’ possible through engagement with the arts, and if so, to establish what form this may take?**

There wasn't a specific question on this topic in our follow-up workshops questionnaire. However, the original participants did articulate a sense of relief in feeling they were not alone with their feelings. Some of the free text comments in the post workshop questionnaire did address the need to support new mothers and the value of a group experience is implicit in some of the remarks about support. One respondent felt that the group experience would be less intimidating that one-to-one therapy and that in “a group you can find connections and belonging, versus feeling alone in your experiences of grief”.

● **What, in particular, does an arts-based approach offer in exploring birth experiences and the transition to motherhood?**

In *Birth Shock!* participants reflected on the ways in which arts-based approaches might be useful for exploring discourses around birth trauma, and their own professional roles, as part of continuing professional education and development, as well as being an integrated part of postnatal maternity service provision - see also Hogan 2021 [5].

The majority of participants reported somewhat agreeing (43.2%) or strongly agreeing (39.8%) with the statement ‘I feel emotionally moved by the films.’ Although it is a small minority, a total of 7.3% of participants strongly or somewhat disagreed with feeling emotionally moved by the films. Across the different professional groups, the mean scores indicated a cluster of similar responses, with midwives (4.1) and student midwives (4.1) scoring similar mean responses to the films, and health visitors (3.4) scoring lower. Obstetricians reported the lowest mean score (2.7), while medical students (4.6) reported the highest incidence of feeling emotionally moved. This data suggests that these films might be particularly emotionally powerful as training resources for students.

## 3 Results

A number of structured workshops aimed at health-care professionals and trainees (28) collated audience responses quantitatively and in free text. The majority of film viewers felt emotionally moved by the films (83%) and a majority felt that watching the films had increased their understanding of antenatal and postnatal practices as a result of the session (58.7%) and in some cases this was a profound and revelatory shift in perspective. Other, more experienced practitioners, (around 23%), were neutral, as felt they were well aware of the issues portrayed, though positively a number reported that the film and discussion session consolidated their *determination* to give good compassionate and patient-centred care. A large number of respondents (88.2%) reported that they are aware that hospital practices are implicated in postnatal distress. A high proportion felt empowered to change their current or future practice as a result of the training session (72.4%), which is a highly noteworthy result. Finally, the vast majority of viewers (89.3%) thought that the project resources (films) would be a useful addition to healthcare training, with a minority of more experienced respondents, feeling they were already au-fait with the issues portrayed.

The films were made available freely so we do not have absolute statistics for their uptake, because they can be downloaded and screened multiple times, we can note the following: that the documentary has had 4.1K views and the film on research methods has had 5.7K views (at November 2022) comparing favourably with academic paper readership estimates. We expect these figures to continue to rise, as the films get embedded into training courses.

## 4 Conclusions

These films have enabled trainees and groups of practitioners to discuss a range of issues pertaining to our central questions. They have raised awareness of the usefulness of arts-based support for new mothers. The films also raise crucial questions about the ethics of practice, questions of consent, dignity in care, and explore how hospital practices are potentially implicated in postnatal distress. Feedback confirms that the resources will be useful in healthcare training. Qualitative feedback indicates that the films are of more value to less experienced practitioners and trainees. General public audiences have also reported being moved by the films. We hope this short communication will further enhance uptake of these resources. The films have also been made available freely via the University of Derby website and YouTube. Film summaries have been added to YouTube so that content is explained, making their use easier: https://www.youtube.com/user/DrSusanHogan/videos.

## Declaration of competing interest

There is no conflict of interest.
